# Comparative Pathogenicity of Wildlife and Bovine *Escherichia coli* O157:H7 Strains in Experimentally Inoculated Neonatal Jersey Calves

**DOI:** 10.3390/vetsci5040088

**Published:** 2018-10-15

**Authors:** Elizabeth M. Antaki-Zukoski, Xunde Li, Patricia A. Pesavento, Tran H. B. Nguyen, Bruce R. Hoar, Edward R. Atwill

**Affiliations:** 1 Department of Population Health and Reproduction, University of California, Davis, CA 95616, USA; emantaki@ucdavis.edu (E.M.A.-Z.); xdli@ucdavis.edu (X.L.); tphnguyen@ucdavis.edu (T.H.B.N.); 2 Western Institute for Food Safety and Security, University of California, Davis, CA 95618, USA; 3 Department of Pathology, Microbiology, and Immunology, University of California, Davis, CA 95616, USA; papesavento@ucdavis.edu; 4 College of Agriculture and Natural Resources, University of Wyoming, Laramie, WY 82071, USA; brucehoar@gmail.com

**Keywords:** Interspecies transfer, intraspecies transfer, bovine, wildlife, *Escherichia coli* O157:H7

## Abstract

Shiga toxin-producing *Escherichia coli*, like *E. coli* O157:H7, are important human and animal pathogens. Naturally-acquired *E. coli* O157:H7 infections occur in numerous species but, particularly, cattle have been identified as a significant reservoir for human cases. *E. coli* O157:H7 are isolated from a number of domestic and wild animals, including rodents that share a living space with cattle. These Shiga toxin-producing *E. coli* O157:H7 strains can be highly virulent in humans, but little is known about the sequelae of interspecies transfer. In a group of neonatal calves, we determined the differences in colonization patterns and lesions associated with infection using either a wildlife or bovine *E. coli* O157:H7 strain. In calves challenged with the wildlife *E. coli* O157:H7 strain, the large (descending) colon was solely colonized, which differed substantially from the calves inoculated with the bovine *E. coli* O157:H7 strain, where the spiral colon was the principal target of infection. This study also demonstrated that while both interspecies- and intraspecies-derived *E. coli* O157:H7 can infect young calves, the distribution and severity differs.

## 1. Introduction

Enterohaemorrhagic *Escherichia coli* comprise a group of emerging zoonotic microorganisms that can be pathogenic, particularly serotype O157:H7. In humans, Shiga toxin-producing *E. coli* (STEC) O157:H7 causes acute gastroenteritis, bloody diarrhea, and hemorrhagic colitis. Approximately 8% of infected individuals can develop hemolytic uremic syndrome (HUS), which can lead to systemic complications or death. The severity of clinical sequelae in STEC *E. coli* O157:H7 infections is commonly linked to the expression and translocation of the bacteriophage-encoded Shiga-toxin across the gut epithelium. Sources of human infection include contaminated foods, primarily ground beef, raw milk, leafy green produce, and even waterborne transmission in rural areas [1,2,3]. 

Neonatal animals, such as calves, are the most susceptible age class for these O157:H7 infections, which cause clinical diarrhea and result in attaching/effacing (A/E) lesions which are severe enterocolitis with fibrinous exudation. Lesions are attributed in part to the attachment of bacterial-derived intimin to the intestinal epithelium. The attachment of *E. coli* O157:H7 to the host’s microvillous border also reduces the tight junction integrity and causes malabsorption and maldigestion from a combination of the loss of both villous enterocytes and microvilli, resulting in villous contraction and the movement of immature crypt cells over the ulcerated surface epithelium [4,5]. 

Naturally-acquired *E. coli* O157:H7 infections occur in numerous species, but cattle have been identified as a significant reservoir for human infections, which is a particularly insidious problem when the animal is asymptomatic. *E. coli* O157:H7 has also been isolated from a number of other domestic and free-range (wildlife) animals, including rodents, which share space with cattle. Nielsen et al. [6] found a low prevalence of Shiga toxin-producing *E. coli* in wild animals living in close proximity to Danish cattle farms but isolates from a starling and a rat had identical serotypes, virulence profiles, and pulsed-field gel electrophoresis types to cattle isolates from corresponding farms, suggesting a possible role in pathogen transmission. Kilonzo et al. [7] also investigated the role that rodents may play in the spread of zoonotic microorganisms among agricultural farms. They concluded that the most abundant rodent species was the deer mouse, where *Cryptosporidium* spp., *Giardia* spp., *Salmonella enterica* serovars, and *E. coli* O157:H7 were isolated from trapped rodent fecal material.

To reduce the risk of *E. coli* O157:H7 transmission from animals to humans, its necessary to understand whether the passage of *E. coli* O157:H7 infections in different animals alter the bacteria’s pathogenicity. Several analytical methods have been able to detect differences/similarities in the strain composition (focusing mainly on intimin types and Shiga-toxin gene profiles) of non-human animal *E. coli* O157:H7 isolates compared to clinical isolates that have infected humans [8]. It is still not well understood why some *E. coli* O157:H7 isolates “adapt” to certain intestinal environments and persist without causing disease, while others can cause a life-threatening condition. Heithoff et al. [9] investigated how particular strains of *Salmonella enterica* spp. emerge and express traits that result in increased virulence or hypervirulence due to passage through certain hosts and/or exposure to environmental variables. This reflects the complexity of the bacterial-host-environment interaction [8,9,10]. Here, we examine the disease progression in neonatal Jersey calves inoculated with strains of *E. coli* O157:H7 with similar virulence composition but isolated from separate animal sources. The objective was to observe lesion and colonization differences between the two *E. coli* O157:H7 isolates. The resultant lesions were examined and scored for intensity, distribution, and percent of intestinal tissue affected to determine the primary colonization sites of each strain. 

## 2. Materials and Methods 

### 2.1. Standard Growth Curve of Bacterial Strains

The wildlife *E. coli* O157:H7 strain was isolated in November 2009 from a deer mouse (*Peromyscus maniculatus*) in Monterey County, CA on a spinach produce ranch. The bovine *E. coli* O157:H7 strain was isolated in July 2007 from a herd of beef cattle at the University of California Sierra Foothill Research and Extension Center in Yuba County, CA. Three growth curves were made for each strain by incubating a bead of stock inoculum, taken from −80 °C frozen microbanks, in 150 mL of Brain Heart Infusion (BHI; Sigma-Aldrich, St. Louis, MO, USA) broth. Each *E. coli* O157:H7 solution was shaken (100 rpm) for 6 h at 37 °C while checking optical density every 30 min with a spectrophotometer (Shimadzu, Torrance, CA, USA) at 610 nm. Serial dilutions from 10^−1^ to 10^−10^ were made in 9 mL of Phosphate Buffered Saline, PBS (Sigma-Aldrich, St. Louis, MO, USA). Dilutions were spread-plated for growth on Luria-Bertani (LB) agar (Sigma-Aldrich, St. Louis, MO, USA) and incubated overnight at 37 °C. Using the number of colonies that grew from each dilution, the overall concentration at each time point was calculated. Each strain was also tested using end-point PCR (Eppendorf, Hauppauge, New York, NY, USA) for Shiga toxins, intimin (*eaeA*), and hemolysin A (*HlyA*) virulence factors (data not shown) [11].

### 2.2. Ethics Statement

All animal experiments were conducted under the approval by Institutional Animal Care and Use Committee (IACUC) of the University of California (UC) Davis Animal Care and Use program, AUP #15459. Experimental procedures followed the federal guidelines outlined in the “Animal Welfare Act” and “Health Research Extension Act,” where personal protective equipment for each pathogen, standard operating protocols for pathogens used, daily cleaning/observation/animal enrichment, and sedation/euthanasia/necropsy methods were described in detail for the review and approval by the committee. 

### 2.3. Animal Experiments

Seven calves approximately 1–2 days old that received colostrum were purchased from an approved dairy. Each calf was housed individually in a biosafety level-II animal facility at the Teaching and Research Animal Care Services (TRACS). The calves were fed commercial milk replacer without antibiotics twice daily and provided free access to water. Bedding material was removed while pens were bleached and rinsed daily, to ensure fresh fecal collection. Upon arrival, each calf was deemed healthy by clinical examination and hematological analysis with a serum chemistry panel and Complete blood count (CBC). Additionally, each calf was tested for the presence of *Cryptosporidium* spp. with an acid-fast stain on individual fecal samples. In order to confirm that calves were not colonized with *E. coli* O157:H7, fresh fecal samples and rectoanal mucosal swabs (RAMS) were collected for three consecutive days prior to experimental inoculation with *E. coli* O157:H7. Ten grams of each fecal sample was measured into 100 mL of Tryptic Soy Broth, TSB (Sigma-Aldrich, St. Louis, MO, USA), while the RAMS were placed in 50 mL of TSB. The samples were incubated at 25 °C for 2 h, 42 °C for 8 h followed by immunomagnetic separation (IMS) using anti-O157 antibodies (Dynal Inc, Camarillo, CA, USA) and cultured on MacConkey II agar with sorbitol, cefixime, potassium tellurite (Becton, Dickinson, Co, Sparks, MD, USA), and Rainbow agar (Biolog, Hayward, CA, USA) for the presence of *E. coli* O157:H7. If any colonies were considered *E. coli* O157:H7 suspects, traditional PCR was performed using a set of specific primers to detect O-antigen-encoding *rfb* regions of *E. coli* O157:H7 [11]. Once the calves were confirmed negative for *Cryptosporidium* and *E. coli* O157:H7 prior infections, they were placed in individual pens, separated by one pen length, and housed in a biosafety level-II facility. They were then assigned an *E. coli* O157:H7 inoculum strain. Throughout the experiment, gowns, gloves, and boot covers were changed between individual pens to prevent cross-contamination.

### 2.4. Inoculation of Animals

One microbank (Pro-lab, Richmond Hill, OH, USA) bead for each strain, stored at −80 °C, was placed in a 250 mL flask containing 150 mL of BHI. The flasks were incubated at 37 °C for 6 h, while shaking at 100 rpm. Once turbidity was observed, the flasks were placed on ice for 15 min. The optical density was then measured at 610 nm with the spectrophotometer and the concentration of the stock solution was calculated by using the generated growth curve from each *E. coli* O157:H7 strain. From the known stock solution concentration, the inoculums were prepared for each strain in PBS. For this study, three calves were inoculated with approximately 10^10^ Colony forming unit (CFU) of the wildlife strain and three calves were inoculated with approximately 10^10^ CFU of the bovine strain, leaving one calf as the negative control. Serial dilutions of the inoculum from 10^−1^ to 10^−10^ were made in 9 mL of PBS and plated in triplicates on LB agar for each stock solution and inoculum. After overnight incubation at 37 °C, the true concentration of each dilution was calculated. The individually-housed calves were intragastrically inoculated with their assigned *E. coli* O157:H7 strain with a calf gastric feeder (VetOne, MWI Veterinary Supply Co., Boise, ID, USA). Each was first given the 200 mL of PBS containing the bacteria and then another 200 mL of PBS as a wash. 

### 2.5. Daily Sampling and Fecal Scoring

Fecal samples and rectoanal mucosal swabs (RAMS) were collected at 24, 48, and 76 h post-inoculation (p.i.). Fecal consistency was scored on a scale from 1–4, with 1 = normal, 2 = paste, 3 = liquid, and 4 = water. All of the samples were collected in the afternoon and transported back to the laboratory in a refrigerated state. The samples were processed within 24 h of collection.

### 2.6. Analytical Assays for Detecting E. coli O157:H7

Each 10 g sample and RAMS were placed in separate Whirl Pak^®^ (eNasco, Fort Atkinson, WI, USA) bags containing 90 mL TSB for fecal samples and 50 mL TSB for RAMS, and then incubated for 2 h at 25 °C, 8 h at 42 °C and held overnight at 6 °C. *E. coli* O157:H7 was recovered using IMS and the Dynal Bead Retriever (Dynal Inc, Camarillo, CA, USA), with 50 μL of washed beads streaked for isolation on Rainbow agar and another 50 μL streaked for isolation on MacConkey II w/Sorbitol Agar. As performed in our laboratory, this IMS method has been shown to detect as few as 1 colony-forming unit (CFU)/10 g of calf feces Two suspect colonies per positive plate were confirmed with end-point PCR [12]. Confirmed colonies were stored at −80 °C in Microbank vials for further analysis.

### 2.7. Euthanasia and Necropsy

One calf from each *E. coli* O157:H7 strain was euthanized with pentobarbital sodium (Euthasol solution) intravenously and necropsied at 24, 48, and 76 h post-inoculation. Intestinal sections of the esophageal groove, abomasum, duodenum, jejunum, ileum, cecum, spiral colon, proximal colon, distal colon, and rectum were collected aseptically for both bacterial culture and histopathology. Sections of pancreas, liver, mesenteric lymph node, gall bladder, lung, and kidney were also taken for both procedures. Each intestinal segment was tied with string by both ends so that the contents and the mucosa could be cultured for the presence of any colonized *E. coli* O157:H7. Once transported to the laboratory, one end of the tissue segment was cut and the contents were collected in a Whirl Pak bag containing 90 mL of TSB. The intestinal tissue was then cut longitudinally to expose the mucosa and placed in a separate Whirl Pak with 90 mL of TSB. The non-intestinal tissues were placed in a bag containing 50 mL of TSB. All TSB bags were then incubated for 2 h at 37 °C, 8 h at 42 °C, and then held overnight at 6 °C. *E. coli* O157:H7 was recovered using the same method as described above. Intestinal and soft tissue sections for histopathology were placed in 10% neutral buffered formalin, provided by the California Animal Health and Food Safety necropsy floor, and allowed to fix for a minimum of two days before trimming.

### 2.8. Histopathology and Lesion Scoring

Fixed tissues were routinely processed, embedded in paraffin, sectioned, and stained with hemotoxylin and eosin (H&E). Histology slides were then examined for pathological changes in each of the tissue segments, which included the state of mucosa (blunted villi, ulceration, and inflammation), adherent bacteria, enumeration of goblet cells, and crypt length. Representative paraffin sections were cut and stained with either a Warthin-Starry Silver stain or Giemsa stain, to ensure that estimation of bacterial load and localization by H&E was accurate. Additional sections stained with Periodic Acid-Schiff (PAS) stain identified the changes in goblet cell number or distribution. The percentage of total area affected was also determined by counting ten glands and calculating the ratio of affected to non-affected glands due to the *E. coli* O157:H7 bacteria. The percentage of glands affected and overall pathological changes (lesions) were then scored on a scale from 1 to 4, where 1 = intact mucosa, 2 = multifocal points of erosion, 3 = bacterial attachment to the surface of the mucosa and in the submucosa with inflammatory cells (i.e., transmigrating neutrophils and eosinophils), and 4 = severe ulceration/mucosal loss with submucosa bacteria with inflammatory cells [13]. Crypt lengths (µm) were measured from proximal and distal colon tissue segments at ten random glands and then averaged to compare to the negative control gland lengths. The tissues from each group of calves were then ranked from most to least affected depending on the overall evaluation score [13]. 

### 2.9. Statistical Analysis

One-way ANOVA tests were performed with SPSS (IBM Corporation, Armonk, New York, USA) computer software on mean crypt lengths from the ten measured glands in the proximal and distal colon at each time increment for the two *E. coli* O157:H7 strains. Additional post hoc tests with Tukey’s multiple-comparison procedure and nonparametric Bonferroni (Dunn) were performed in order to compare each mean against the negative control crypt length means. All tests were performed at a level of 0.05. 

## 3. Results

Both the wildlife and bovine *E. coli* O157:H7 strains encoded Shiga toxins I and II, intimin (*eaeA*), and hemolysin A (*HlyA*) genes. Diarrhea progressed over three days in both groups of inoculated calves. Despite intensive pre-screening, four out of the six inoculated calves were concurrently infected with *Cryptosporidium* spp., which was taken into consideration during pathological evaluation and scoring. These included, calves inoculated with the wildlife *E. coli* O157:H7 strain euthanized at 48 and 76 h post-inoculation; calves inoculated with the bovine *E. coli* O157:H7 strain euthanized at 24 and 76 h post-inoculation

The uninoculated control calf used for this experiment was sacrificed and presented no clinical signs of diarrhea, no gross lesions, and no histopathological evidence of intestinal disease. In all inoculated cases, regardless of the strain of *E. coli* O157:H7 and time post-inoculation, the lesions associated with attached *E. coli* O157:H7 included mucosal attenuation, erosion, ulceration, necrosis, edema, and bacterial presence in the submucosa. These changes only occurred in the large intestinal segments. Lesions for *E. coli* O157:H7 have been previously described [14,15,16]. The group of calves inoculated with the wildlife *E. coli* O157:H7 strain had submucosal bacteria present in at least one segment of large intestine at all post-inoculation time points, where the calf inoculated with the bovine *E. coli* O157:H7 stain euthanized 76 h post-inoculation had submucosal bacteria present in the cecum and spiral colon sections only. 

**Figure 1:**
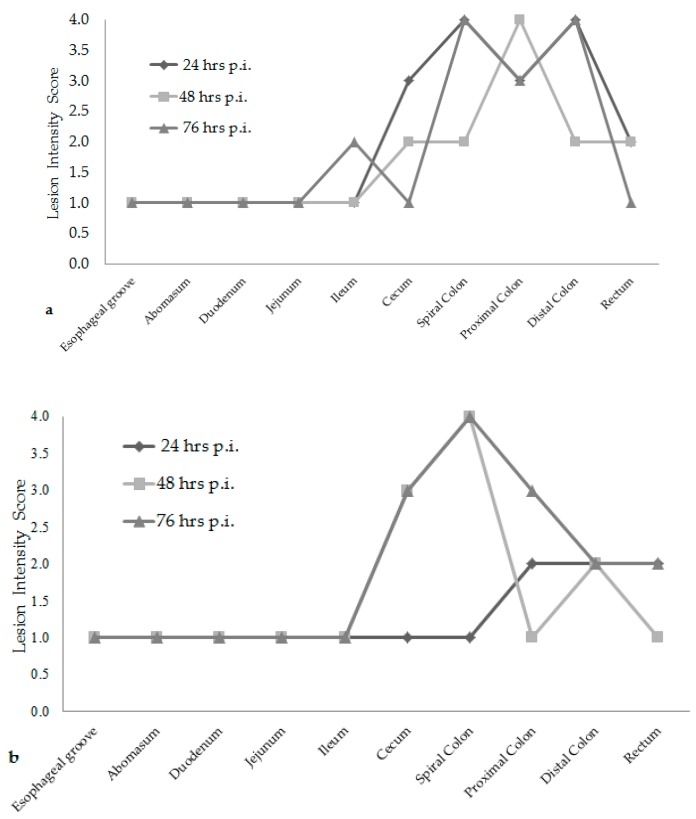
Overall lesion scores at each time increment for the wildlife *E. coli* O157:H7 inoculum group of calves (**a**). Overall lesion scores at each time increment for the bovine *E. coli* O157:H7 inoculum group of calves (**b**). (hrs) hours; (p.i.) post-inoculation.

**Figure 2:**
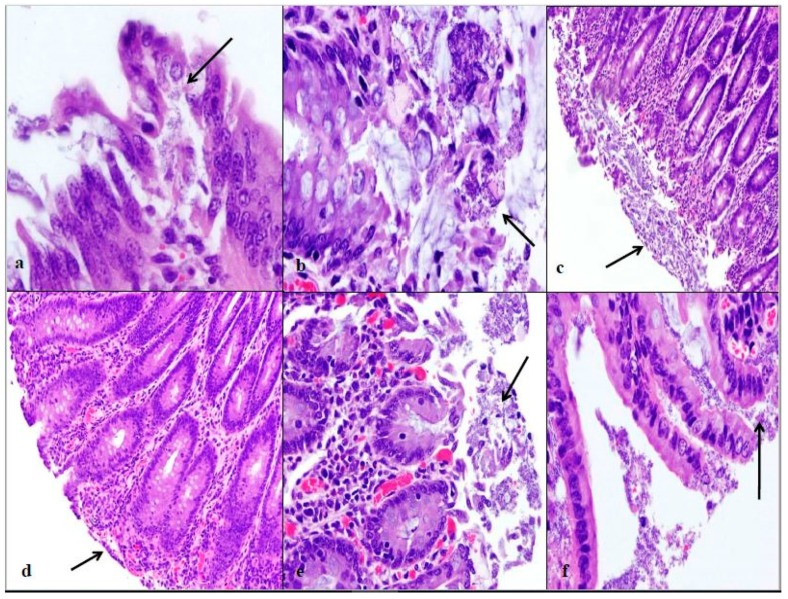
Intestinal lesion comparison of wildlife and bovine *E. coli* O157:H7 at each euthanasia time increment. Wildlife *E. coli* O157:H7 at 24 h post-inoculation contains mucosal exfoliation and attaching/effacing lesion formation in the spiral colon at 40× (**a**). Wildlife *E. coli* O157:H7 at 48 h post-inoculation contains edema, exfoliation of mucosa, and mucus secretion in the proximal colon at 40× (**b**). Wildlife *E. coli* O157:H7 at 76 h post-inoculation contains ulceration, exfoliation, and a large area of necrotic lesion in the spiral colon at 10× (**c**). Bovine *E. coli* O157:H7 at 24 h post-inoculation has attenuated and blunted mucosal epithelium in the distal colon at 10× (**d**). Bovine *E. coli* O157:H7 at 48 h post-inoculation contains multifocal *E. coli* bacteria and dilated crypt glands in the spiral colon at 20× (**e**). Bovine *E. coli* O157:H7 at 76 h post-inoculation contains necrotic mucosa and submucosa *E. coli* bacteria in the spiral colon at 40× (**f**). The arrows indicate the pathological changes at each time point. These sections were all stained with hemotoxylin and eosin (HE).

**Figure 3:**
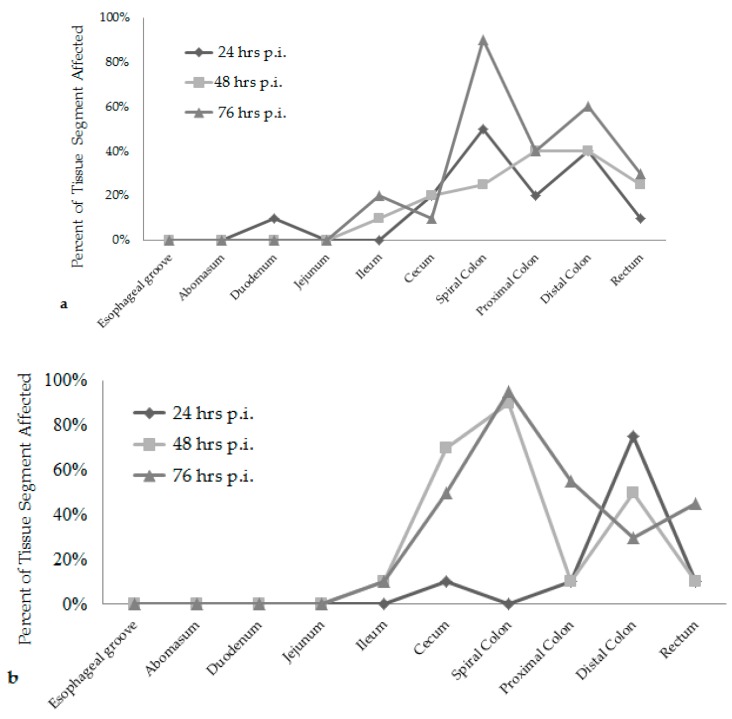
Percent of the total intestinal tissue segment affected at each time increment for the wildlife *E. coli* O157:H7 inoculum group of calves (**a**). Percent of the total intestinal tissue segment affected at each time increment for the bovine *E. coli* O157:H7 inoculum group of calves (**b**). (hrs) hours; (p.i.) post-inoculation.

Attaching/effacing (A/E) lesion development was different among the time increments after inoculation. Lesion scores were the greatest at 76 h post-inoculation for both *E. coli* O157:H7 inoculum groups, see Figure 1. The wildlife and bovine *E. coli* O157:H7 inoculum groups had very different lesion distribution and severity. With the progression of time post-inoculation, the calves inoculated with the wildlife *E. coli* O157:H7 strain had over 20% of their large intestinal segments affected by bacterial presence and lesion development. The calf euthanized at 24 h post-inoculation had 40–50% of necrotic mucosa in its spiral colon and distal colon sections, with transmigrating neutrophils and multifocal bacterial attachment. This progressed to 90% spiral colon and 60% distal colon containing necrotic mucosa and crypt dilation in the calf euthanized at 76 h post-inoculation. Lesions progressed much more rapidly in the group inoculated with the bovine *E. coli* O157:H7 strain, with 75% of necrotic mucosa and a focal area of bacterial attachment in the distal colon of the calf euthanized at 24 h post-inoculation. The distribution and severity of affected intestinal tissue then changed in the bovine *E. coli* O157:H7 inoculum group. In calves euthanized at 48 and 76 h post-inoculation, 90% of the spiral colon mucosa was affected by diffuse bacterial attachment to the surface and submucosa. These changes can be seen histologically in Figure 2a–f. Overall, the wildlife *E. coli* O157:H7 strain moderately affected the entire large intestine, while the bovine *E. coli* O157:H7 strain mainly colonized the spiral colon, see Figure 3. 

After ANOVA and post hoc testing, mean crypt lengths in the proximal colon were not significantly different (*p* = 0.109) when each *E. coli* O157:H7 group was compared by time increments, but were significantly different (*p* < 0.001) when compared within each *E. coli* O157:H7 inoculum group. The 24 h post-inoculation mean crypt lengths in the wildlife and bovine *E. coli* O157:H7 calves were both longer when compared to the calves euthanized at 48 h post-inoculation, who had mean lengths of 104.3 µm and 90.1 µm. All mean lengths were significantly longer when compared to the negative control mean, except for the calf inoculated with the bovine *E. coli* O157:H7 strain euthanized at 48 h post-inoculation. When comparing within *E. coli* O157:H7 inoculum groups for the distal colon, the calves inoculated with the wildlife strain had no significant differences (*p* = 0.287) in mean lengths but the calf inoculated with the bovine strain euthanized at 24 h post-inoculation had significantly longer crypt lengths when compared to the calves inoculated with the bovine strain euthanized at 48 and 76 h post-inoculation (*p* = 0.004, *p* < 0.001). There was also a significant difference in mean lengths at 48 and 76 h post-inoculation when each *E. coli* O157:H7 group was compared by time increments (*p* = 0.037 and *p* = 0.004). For the distal colon, the negative control mean length was only significantly shorter when compared to the calf inoculated with the bovine strain euthanized at 24 h post-inoculation (*p* < 0.001) and the calf inoculated with the wildlife strain euthanized 48 h post-inoculation (*p* = 0.003), see Table 1.

## 4. Discussion

This study was a temporal analysis of lesion progression that compares wildlife and bovine *E. coli* O157:H7 strains in calves. Due to space and resources, only one calf represented each inoculum strain at the three post-inoculation time points. The high inoculum dose was to ensure that an infection would occur. Previous ID_50_ (infectious dose at 50%) trials in calves with other strains of *E. coli* O157:H7 (data not published), showed that a high inoculum dose was needed to produce and sustain an infection in this animal model. While working with the strains in the lab, no significant differences in growth curves were seen. The main difference was the host that the strain was isolated from. It was hypothesized that the interspecies inoculation, a wildlife *E. coli* O157:H7 strain into calves, would result in more severe lesion development than intraspecies inoculation. Alternatively, some reports have demonstrated that the horizontal transmission of certain bacteria among animals of the same species changes the infection pattern, allowing the pathogen to adapt to the host’s intestinal environment and giving it the ability to translocate to other tissues or become “hyperinfectious” [17,18,19,20]. In this study, the wildlife *E. coli* O157:H7 strain moderately affected the entire large intestine, colonizing the spiral colon, proximal colon, and distal colon evenly and then affecting the cecum and the rectum. This was in comparison to the bovine *E. coli* O157:H7 strain which primarily colonized the spiral colon, then cecum, proximal colon, and distal colon. The “adaptive” characteristic of the bovine-bovine passage in this study, where the bovine *E. coli* O157:H7 strain severely affected the spiral colon of the inoculated calves, could be associated with specific genes being upregulated or downregulated due to the intraspecies transmission or using an *E. coli* O157:H7 isolated from naturally infected cattle that produced a low infection [10]. In each of the intestinal tissues, bacteria (presumably inoculated *E. coli* O157:H7) were seen closely attached to the surface of the enterocytes, with many affected epithelial cells in the process of necrosis and moving into the intestinal lumen. Some intestinal areas were so necrotic that bacteria were seen invading the submucosa. 

There was also a difference in the infection patterns of the *E. coli* O157:H7 strains at each time increment after inoculation. By increasing the length of time between inoculation and necropsy from 24 h to 76 h, it was demonstrated that both *E. coli* O157:H7 strains induced extensive A/E lesions [2]. At 24 h post-inoculation, the wildlife strain caused more lesions throughout the large intestine compared to the bovine strain, shown in Figure 1 and Figure 3, with lesion scores and percent of each intestinal segment affected. The bovine *E. coli* O157:H7 group had only small focal points of bacterial attachment in the proximal colon and rectum with intact mucosa, but the distal colon had segmentally necrotic and attenuated mucosa. There was one area of bacterial attachment, but the production of bacterial toxins might account for the 75% mucosal destruction. At 48 and 76 h post-inoculation, both strains began to cause more damage in the affected sections of the large intestine. The progression of lesion formation at these two time points was assisted by the additional silver stain performed on the affected tissue sections. Both *E. coli* O157:H7 strains were able to form A/E lesions, but it was demonstrated that the wildlife strain started to cause damage in the large intestine within the first 24 h after inoculation compared to the bovine *E. coli* O157:H7 strain, which became more damaging around the 48 and 76 h mark.

Measurements of crypt lengths in the proximal and distal colon from the experimentally inoculated calves were evaluated and compared to the negative control crypt lengths to determine the severity of infection and cell turnover. In the proximal colon, all mean crypt lengths were considered significantly different in comparison with the negative control crypt length, except for the calf inoculated with the bovine *E. coli* O157:H7 strain euthanized at 48 h post-inoculation. Longer crypt lengths were observed in the 24 h post-inoculation wildlife and bovine *E. coli* O157:H7 calves when compared to the calves euthanized at 48 h post-inoculation. The distal colon had significant differences within the bovine *E. coli* O157:H7 group when the mean length of the calf euthanized at 24 h post-inoculation was compared to the calves euthanized at 48 and 76 h post-inoculation, as well as among each *E. coli* O157:H7 strain at 48 and 76 h post-inoculation time increments. The negative control mean length was only significantly different when compared to the calf inoculated with the bovine *E. coli* O157:H7 euthanized at 24 h post-inoculation and the wildlife inoculated calf euthanized at 48 h post-inoculation. The difference in crypt lengths of significant comparisons can be attributed to an adaptive hyperplastic response or increased cell turnover due to the mucosal injury which is being caused by the bacterial infection [21,22].

Despite pre-screening of the calves upon arrival, four out of the six inoculated calves were concurrently infected with a *Cryptosporidium* spp. infection present in the ileum. It is possible that the compromise or morbidity from the *E. coli* O157:H7 infection made the calves exquisitely sensitive to an inoculum of *Cryptosporidium* spp., prior exposure or contracted during the study, that was undetected by our diagnostic assays [23]. No evidence of a *Cryptosporidium* spp. infection was seen in the control calf. 

In this study, a wildlife *E. coli* O157:H7 isolate from a deer mouse was demonstrated to be pathogenic and was capable of forming A/E lesions throughout the entire large intestine of neonatal calves in comparison to a bovine *E. coli* O157:H7 isolate that intensely affected one area of the large intestine. It was important to use this wildlife strain since wild mice can potentially carry *E. coli* O157:H7, causing possible transmission to humans when coming in contact with farm animals and produce fields [24]. Both *E. coli* O157:H7 strains have similar virulence capabilities to form A/E lesions but have different tissue infection patterns and severity in neonatal calves, possibly due to inter and/or intraspecies interactions with the host. Future studies will help investigate these relationships further with various inter and intraspecies hosts to determine the change in virulence patterns between various *E. coli* O157:H7 isolates.

## 5. Conclusions

Both strains of *E. coli* O157:H7, isolated from different animal sources, were shown to be pathogenic in this calf model, where one was more severe with regard to intestinal tissue colonization in comparison to the other. The wildlife *E. coli* O157:H7 strain was able to affect a wider distribution of the large intestine when compared to the bovine *E. coli* O157:H7 strain, which mainly affected the spiral colon. Due to the infection, crypt lengths within the proximal and distal colon were different in length when compared to the negative control, meaning that an adaptive response was occurring due to the mucosal injury. It was significant to show the lesion progression and differences in the pathogenesis of both *E. coli* O157:H7 strains over a 76 h period since *E. coli* O157:H7 can be easily transmitted to a wide range of hosts. Even though clinical signs may be slight or not present, infections can still be occurring, leading to potential contamination.

## Figures and Tables

**Table 1:** Comparison of mean crypt lengths (µm) of ten glands post-inoculation of the (a) proximal colon and (b) distal colon at each time increment for each inoculated *E. coli* O157:H7 group using Tukey’s multiple-comparison procedure.

a		
**Time (h post-inoculation)**	**Wildlife (µm)**	**Bovine (µm)**
24	125.0 ^A^	114.0 ^A,B^
48	104.3 ^B, C^	90.1 ^C, D^
76	108.7 ^A, B^	106.4 ^B,C^
**Negative control value** = 85.6 ^D^		
b		
**Time (h post-inoculation)**	**Wildlife (µm)**	**Bovine (µm)**
24	96.7 ^A,B^	116.3 ^A^
48	111.4 ^A^	90.7 ^B,C^
76	101.8 ^A,B^	76.4 ^C^
**Negative control value** = 85.3 ^B,C^		

Means without a superscript in common are statistically significantly different with a level of significance of 5% over all comparisons using Tukey’s multiple-comparison procedure.
